# Evaluation of dipstick analysis among elderly residents to detect bacteriuria: a cross-sectional study in 32 nursing homes

**DOI:** 10.1186/1471-2318-9-32

**Published:** 2009-07-27

**Authors:** Pär-Daniel Sundvall, Ronny K Gunnarsson

**Affiliations:** 1 Sandared Primary Health Care Centre, Sandared, Sweden; 2 Research and development unit, Primary Health Care in Southern Älvsborg county, Älvsborg, Sweden; 3 Department of Public Health and Community Medicine, Institute of Medicine, Sahlgrenska Academy at University of Gothenburg, Gothenburg, Sweden

## Abstract

**Background:**

Few studies have evaluated dipstick urinalysis for elderly and practically none present confidence intervals. Furthermore, most previous studies combine all bacteria species in a "positive culture". Thus, their evaluation may be inappropriate due to Yule-Simpson's paradox. The aim of this study was to evaluate diagnostic accuracy of dipstick urinalysis for the elderly in nursing homes.

**Methods:**

In this cross-sectional study voided urine specimens were collected from 651 elderly individuals in nursing homes. Dipstick urinalysis for nitrite, leukocyte esterase and urine culture were performed. Sensitivity, specificity, positive and negative predictive values with 95% confidence intervals were calculated. Visual readings were compared to readings with a urine chemistry analyzer.

**Results:**

207/651 (32%) of urine cultures showed growth of a potentially pathogenic bacterium. Combining the two dipsticks improved test characteristics slightly compared to using only one of the dipsticks. When both dipsticks are negative, presence of potentially pathogenic bacteria can be ruled out with a negative predictive value of 88 (84–92)%. Visual and analyzer readings had acceptable agreement.

**Conclusion:**

When investigating for bacteriuria in elderly people at nursing homes we suggest nitrite and leukocyte esterase dipstick be combined. There are no clinically relevant differences between visual and analyzer dipstick readings. When dipstick urinalysis for nitrite and leukocyte esterase are both negative it is unlikely that the urine culture will show growth of potentially pathogenic bacteria and in a patient with an uncomplicated illness further testing is unnecessary.

## Background

Urinary Tract Infection (UTI) is the most common bacterial infection among elderly residents of nursing homes [1] and often results in antibiotic treatment [2]. Thus, a correct diagnosis is important for minimizing unnecessary antibiotic treatment. Dipstick urinalysis is often the first measure for detecting bacteriuria [3]. The diagnostic value of dipstick urinalysis is most often evaluated for children and working age adults, preferably women which may lead to different results depending on age group and patient criteria. Thus, the clinical value of dipstick urinalysis could be quite different for elderly patients at nursing homes compared to younger patients whereby elderly patients have a higher prevalence of bacteriuria [1,4,5].

Numerous errors can occur during the testing procedure of urine dipsticks [6]. Timing and misalignment errors as well as subjectivity can be reduced by using a urine chemistry analyzer and thus achieve better precision [6-8]. Other studies showed only minor improved reproducibility [9,10] and no improvement in speed of analysis [10] by using mechanized methods. Furthermore, when urine tests are performed under daily conditions results can be considerably lower, even for simple tests such as nitrite, than for optimal and standardized conditions achieved in most studies of the validity of urine tests [11]. Thus, the importance of analyzer readings compared to visual readings of dipsticks in nursing homes for elderly remains to be clarified.

It should be noted that while sensitivity and specificity are of major interest for manufacturers of dipsticks these measures are of no interest to the physician making a clinical decision in one case. The positive predictive value (PPV) and the negative predictive value (NPV), however, are of the utmost clinical importance to the physician. These values are affected by the prevalence of bacteriuria [12].

When estimating sensitivity and specificity it is appropriate to present an interval estimate [13,14]. This is rarely done in studies evaluating diagnostic tests [13]. The precision of predictive values, as with sensitivity and specificity, is dependent on the sample size [13]. It is therefore also appropriate to use some kind of interval estimate for predictive values.

Unfortunately, only one previously published study evaluating dipstick urinalysis in elderly has presented confidence intervals for PPV and NPV [15]. Other studies evaluating dipstick urinalysis of the elderly present confidence intervals only for sensitivity and specificity [16,17] or no confidence intervals at all [8,18-23].

Furthermore, as Yule-Simpson's statistical paradox predicts, the outcome of analysing a single bacterium might differ from analysing "any bacteria" [24-26]. In such cases, results from analysing a single bacterium are more appropriate while results of analysing "any bacteria" are inappropriate. All previously published studies evaluating dipstick urinalysis of the elderly combine different bacterium to "any bacteria" when calculating sensitivity, specificity, PPV or NPV.

The primary aim of this study was to document the sensitivity, specificity, PPV and NPV with 95% confidence intervals for detection of bacteriuria among males and females in nursing homes for the elderly by dipstick urinalysis performed by non-laboratory personnel. The secondary aim was to compare manual readings of dipstick urinalysis with a urine chemistry analyzer.

## Methods

A single, voided specimen of urine was collected, the urine dipstick analyzed and the urine cultured from elderly individuals at nursing homes during a four-week period in the first months of 2003. The nursing homes were located in southwestern Sweden. The study was approved by the ethical committee Göteborg University.

### Selection of individuals

Specimens of voided urine were collected from all individuals present at the nursing home agreeing to participate and sufficiently continent to leave a specimen of voided urine. In case of dementia, where the individual did not understand the provided information, a sample was taken only if permission was granted. Those with urinary indwelling catheters were excluded. Urine specimens were from permanent elderly residents of 102 wards in 32 nursing homes.

### Laboratory tests

Personnel were instructed to collect voided, midstream urine specimens with as long a bladder incubation time as possible, preferably a morning sample.

Immediately after urination, dipstick urinalysis was carried out at the nursing home. Visual reading of the urinary dipstick Multistix 5 (Bayer HealthCare Diagnostics Division) was performed first for the detection of nitrite and leukocyte esterase. Then, a second urinary dipstick (also Multistix 5) was analyzed for the detection of nitrite and leukocytes, with the urine chemistry analyzer Clinitek 50 (Bayer HealthCare Diagnostics Division) [27]. The nursing home personnel were instructed by a representative from the manufacturer in the handling of the Multistix 5 and the analyzer Clinitek 50. The attending nurses were carefully instructed to record the results of the visual readings before using the urine chemistry analyzer. Thus, the visual readings were not influenced by the results of the urine chemistry analyzer.

Immediately after the dipstick readings the urine samples were chilled awaiting transport to the microbiological laboratory in Borås where all urine specimens were cultured. The samples usually reached the laboratory within 24 hours.

By using sterile inoculating loops the microbiology laboratory fractionated 10 μl from the urine samples on the surfaces of two plates; a cystine-lactose-electrolyte deficient agar (CLED) and a Columbia blood agar base. Both plates were incubated overnight (minimum 15 h) at 35–37° C. The CLED plates were incubated in air and the Columbia plates were incubated in 5% CO_2_. The latter were further incubated 24 hours if no growth occurred after first incubation.

A culture with growth of potentially pathogenic bacteria was normally considered positive if the number of colony-forming units per liter (CFU/mL) was ≥10^5^. In case of specific signs of possible UTI such as positive nitrite dipstick, leukocyte esterase dipstick >1, fever, frequency, urgency or dysuria, the cut-off point was ≥10^3 ^for patients harbouring *Escherichia coli *and male patients with *Klebsiella *species and *Enterococcus faecalis*. For symptomatic women with the two latter species the cut-off level was ≥10^4^.

### Statistical analysis

Sensitivity, specificity, PPV and NPV were calculated for nitrite and leukocyte esterase separately using urine culture as gold standard. Similar estimates were calculated for combinations of nitrite and leukocytes. Ruling in or ruling out bacteriuria was considered possible where the point estimate of PPV/NPV was ≥ 85% with a lower confidence interval of ≥ 80%.

Agreement between visual and analyzer readings of dipsticks was calculated by Kappa coefficient.

To avoid confounding factors such as sex and age leading to false conclusions the association between dipstick findings and urine cultures was further evaluated by logistic regression. Presence or absence of a potentially pathogenic bacterium in urine culture was the dependent variable while outcome of dipstick, sex and age were independent variables.

Epi Info version 3.3.2 (Windows version) (CDC, Atlanta, USA) was used for storing data and for logistic regression. Calculations for sensitivity, specificity, PPV and NPV were made in Microsoft Office Excel 2003 version 11.8 SP2. Kappa coefficient with confidence intervals was calculated using CIA (Confidence Interval Analysis) version 2.1.2 (Bryant, University of Southampton, England) [28].

## Results

**Figure 1:**
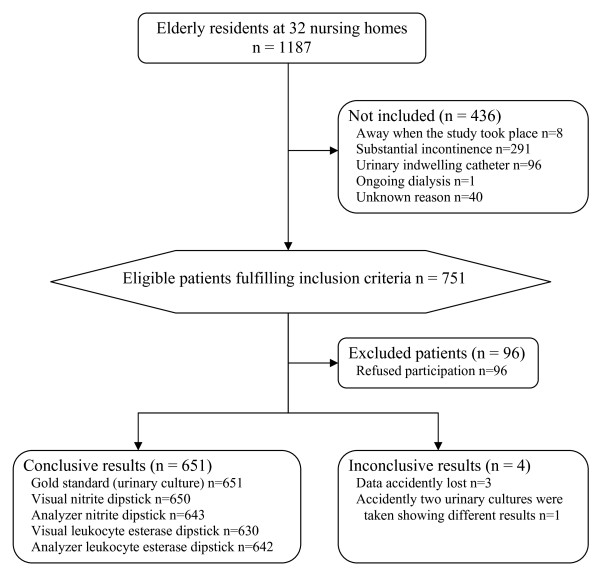
**Participants flow chart**.

There were 1187 individuals living in 32 participating nursing homes (Figure 1). Of the 751 fulfilling inclusion criteria 655 (87%) accepted participation (Figure 1). Of the 651 individuals providing useful samples, 482 (74.0%) were women and 169 (26.0%) men. Women's ages (mean 86 years, SD 7.4, range 46–102) were higher than men's (mean 82 years, SD 7.8, range 54–99) (p < 10^-4^).

### The most common bacterium

In this study 207/651 (32%) urine cultures showed growth of a potentially pathogenic bacterium. The three most common bacteria were *E. coli *(143 = 22%), *Klebsiella *spp. (25 = 3.8%) and *E. faecalis *(17 = 2.6%). Other species had a considerably lower prevalence (≤0.8% for each bacterium).

### Sensitivity, specificity, PPV and NPV

If a single leukocyte esterase dipstick was negative then the high NPV showed it unlikely that the urine culture was positive for *E. coli*, *E. faecalis *and *Klebsiella *spp [see Additional file 1]. However, presence of "any bacteria" could not be excluded [see Additional file 1]. If a single leukocyte esterase dipstick was positive it could not sufficiently predict bacteriuria [see Additional file 1].

Where a single nitrite dipstick was negative the high NPV showed it unlikely that the culture was positive for *E. coli*, *E. faecalis *and *Klebsiella *spp. respectively [see Additional file 2]. However, presence of "any bacteria" could not be excluded [see Additional file 2]. If a single nitrite dipstick was positive it could not sufficiently predict bacteriuria [see Additional file 2].

A single negative nitrite dipstick was as good as a single negative leukocyte esterase dipstick in excluding presence of *E. coli *[see Additional file 1] [see Additional file 2]. Raising the cut-off point in a single leukocyte esterase dipstick decreased its ability to exclude *E. coli *without making PPV for *E. coli *acceptable [see Additional file 2]. Thus, if a single dipstick was to exclude or predict presence of *E. coli *then the nitrite dipstick performed better than the leukocyte esterase dipstick. The accuracy of excluding or predicting *E. faecalis *and *Klebsiella *spp. did not differ between a single leukocyte esterase dipstick or a single nitrite dipstick [see Additional file 1] [see Additional file 2].

Combining the two dipsticks so that presence of both leukocyte esterase **and **nitrite were considered a positive test and all other test outcomes as negative altered test characteristics slightly compared to using only one of the dipsticks [see Additional file 3]. NPV for predicting absence of *E. coli *was lower compared to using only one of the dipsticks while PPV for predicting presence of *E. coli *increased only marginally.

Combining the two dipsticks so that presence of leukocytes **and/or **nitrite were considered positive and all other test outcomes as negative also altered test characteristics compared to using only one of the dip sticks [see Additional file 4]. NPV for predicting absence of a specified potentially pathogenic bacteria or predicting "any bacteria" was high enough to rule out bacteriuria.

### Agreement between visual reading and analyzer

Visual and analyzer readings had, for nitrite, good agreement with a kappa coefficient 0.92 (95% confidence interval 0.88–0.95, SE for kappa 0.019). However, the agreement for leukocyte esterase was lower with kappa coefficient 0.54 (95% confidence interval 0.49–0.60, SE for kappa 0.027).

### Association between dip stick findings and urine culture

The association between dipstick findings and urine culture was further evaluated by logistic regression to consider gender or age dependent effects. A visually read leukocyte esterase dipstick > 0 added information to the question of whether *E. coli *and "any bacteria" were present in the urine [see Additional file 5]. For *E. faecalis *and *Klebsiella *spp. a leukocyte esterase dipstick added information in some color blocks but not in others [see Additional file 5].

A positive nitrite dipstick, visually or analyzer read, added information to the question if *E. coli, Klebsiella *spp. or "any bacteria" was present in the urine [see Additional file 5]. For *E. faecalis *a nitrite dipstick added no statistically significant information [see Additional file 5].

When determining whether *E. coli *or "any bacteria" was present in the urine the use of nitrite dipstick added more information than the use of a leukocyte esterase dipstick [see Additional file 5].

## Discussion

When dipstick urinalyses for nitrite and leukocyte esterase are simultaneously negative it is unlikely that the urine culture will show growth of potentially pathogenic bacteria. There are no clinically relevant differences between visual and analyzer readings of the dipsticks.

### Methodological aspects

In this study we obtained a urine specimen from 55% (651/1187) of all individuals registered at the nursing homes. This may appear low but approximates previously published studies in nursing homes for elderly [4]. The main reason for nonparticipation in this study was substantial urinary incontinence. Most of these individuals also had dementia. The only possibility of obtaining a urine specimen from these individuals would have been by catheter. This is not routine for clinical practice for elderly at nursing homes and would, therefore, not have been representative of clinical practice. Furthermore, this would have been unethical. Individuals with an indwelling urinary catheter were excluded as they always become colonized by bacteria sometimes of different species compared to those without [1]. Only 12% (96/791) actively refused participation which we considered acceptable.

The study by Juthani-Mehta et al [15] presenting confidence intervals for PPV and NPV included only patients with symptoms of suspected UTI. Specific symptoms were dysuria (7%), change in voiding pattern (6%) or fever (12%) and unspecific symptoms were change in mental status (40%), behavior (20%), character of urine (17%), and evaluation for other infection (7%), family or patient request (7%), etc. However, it is unclear which clinical features or events are relevant in bacteriuria [1,29]. Thus, while Juthani-Mehta et al attempt to estimate dipstick analysis ability to predict UTI, this study focused on evaluating dipstick ability to predict bacteriuria, not UTI. Prevalence of bacteriuria among asymptomatic residents in nursing homes for elderly is high [1,4,5] and similar to the prevalence found by Juthani-Mehta et al (40%) and in this study (32%). Since PPV and NPV for dipstick analysis depend on prevalence of bacteriuria there should be no major differences between evaluating dipstick analysis for symptomatic or asymptomatic individuals.

Only 4.0% (26/651) had ongoing antibiotic treatment thus no urinary bacteria growth and negative nitrite dipstick may have been expected. However, leukocyte esterase dipstick may remain positive for some time. Thus, these patients were more likely to influence test results of leukocyte esterase rather than nitrite dipstick. Due to the low prevalence of ongoing antibiotic treatment this effect was considered low.

Procedures allowing presence of a few specific symptoms or outcomes of prior dipstick testing influence the decision of cut-off levels for CFU in urine culture may enhance the diagnostic procedure [30]. These procedures are very common in microbiologic laboratories in Sweden. Thus, the present procedure for urine culture was used without modification to be representative of ordinary clinical practice.

Dipstick urinalysis was performed by non-laboratory personnel in this study. If the analysis had been performed by laboratory personnel, results might have differed slightly. On the other hand, these bedside tests are usually performed by non-laboratory personnel in clinical practice at nursing homes for the elderly. Thus, this study represented ordinary clinical practice.

The results of this study can be considered generalisable in developed countries when evaluating urine dipstick analysis for elderly individuals at nursing homes performed in ordinary clinical practice.

The NPV for nitrite to predict absence of *E. faecalis *in a urine culture is higher than for *E. coli *despite *E. faecalis *being a poor converter of nitrate to nitrite. The most likely explanation being the prevalence of *E. faecalis *is very low (2.6%) resulting in a high NPV even if sensitivity and specificity are low.

The kappa coefficient for agreement between visual and analyzer readings was lower for leukocyte esterase dipsticks than for nitrite dipsticks. This is logical whereby leukocyte esterase dipsticks have several color blocks while nitrite dipstick has only a binary outcome. The more possible outcomes the lower the kappa value becomes.

### The effect of Simpson's paradox when evaluating dipsticks

It may seem peculiar that PPV for any bacteria is higher than PPV for a single bacterium [see Additional file 1] [see Additional file 2] [see Additional file 3] [see Additional file 4]. One explanation is prevalence of bacteria in the gold standard is higher when the focus is on "any bacteria" compared to a specific bacterium subsequently decreasing the probability of a false positive dipstick. The reverse was seen for NPV.

Another way to explain this phenomenon is the well known Simpson's paradox. Several potentially pathogenic bacteria differ in their ability to reduce nitrate to nitrite. Similarly, different bacteria are likely to show a varying ability to provoke pyuria. This difference is a confounding factor and as the prevalence of the different types of bacteria varies considerably the size of these groups vary. This phenomenon has been previously explained as Yule-Simpson's effect, a statistical paradox in which the outcome of several groups is changed when groups are combined [24-26].

The conclusion that when dipstick urinalyses for nitrite and leukocyte esterase are simultaneously negative it is unlikely that the urine culture will show growth of potentially pathogenic bacteria is based on "any bacteria" [see Additional file 4]. Furthermore, NPV for each single bacterium is higher thus the conclusion seems valid. If one should decide that positive nitrite dipstick can rule in bacteriuria, as a previously published metaanalysis did [16], we find that PPV for "any bacteria" differs very much from PPV for the single bacteria [see Additional file 2]. Thus, the conclusion that positive nitrite dipstick can rule in bacteriuria seems unjustified.

## Conclusion

It is beyond the scope of this study to conclude which clinical conditions should be investigated for bacteriuria. However, if there is a reason for investigating this, the present study may provide some simple guidelines.

When testing is performed in nursing homes for elderly by non-laboratory personnel this study showed that there were no clinically relevant differences between visual and analyzer reading of dipsticks. Thus, the choice between visual and analyzer reading could be based on personal preferences and economic aspects.

A single nitrite dipstick performs as well or better than a single leukocyte esterase dipstick. However, different combinations of the dipsticks improve the diagnostic value.

When dipstick urinalyses for nitrite and leukocyte esterase are simultaneously negative it is unlikely that the urine culture will show growth of potentially pathogenic bacteria. Thus, in a patient with an uncomplicated illness, no further testing is needed. However, a positive dipstick or any combination thereof cannot completely rule in bacteriuria.

## Competing interests

The authors declare that they have no competing interests.

## Authors' contributions

PDS and RG planned the study. PDS carried out the data collection, analyses and drafted the manuscript. RG supervised data collection, analyses and the writing of the manuscript. All authors reviewed and approved the final draft of the paper.

## Pre-publication history

The pre-publication history for this paper can be accessed here:



## Supplementary Material

Additional file 1 **Table 1 – Test characteristics of a single leukocyte esterase dipstick compared to urine culture. **Test characteristics, such as sensitivity, specificity, positive and negative predictive value, of a single leukocyte esterase dipstick compared to urine culture. Click here for file

Additional file 2 **Table 2 – Test characteristics of a single nitrite dipstick compared to urine culture. **Test characteristics, such as sensitivity, specificity, positive and negative predictive value, of a single nitrite dipstick compared to urine culture. Click here for file

Additional file 3 **Table 3 – Test characteristics of a positive leukocyte esterase and a positive nitrite dipstick compared to urine culture. **Test characteristics, such as sensitivity, specificity, positive and negative predictive value, of a positive leukocyte esterase and a positive nitrite dipstick compared to urine culture. Click here for file

Additional file 4 **Table 4 – Test characteristics of a positive leukocyte esterase and/or a positive nitrite dipstick compared to urine culture. **Test characteristics, such as sensitivity, specificity, positive and negative predictive value, of a positive leukocyte esterase and/or a positive nitrite dipstick compared to urine culture. Click here for file

Additional file 5 **Table 5 – Odds ratio for a positive dipstick to predict presence of bacteriuria when considering age and sex. **Odds ratio for a positive dipstick to predict presence of bacteriuria when considering age and sex. Click here for file
