# Supplementary material for: Evaluation of dipstick analysis among elderly residents to detect bacteriuria: a cross-sectional study in 32 nursing homes
Source: BMC Geriatr. 2009 Jul 27;9:32. doi: 10.1186/1471-2318-9-32 (PMC2724370; doi:10.1186/1471-2318-9-32)
Supplement: Additional file 1 — Table 1 – Test characteristics of a single leukocyte esterase dipstick compared to urine culture. Test characteristics, such as sensitivity, specificity, positive and negative predictive value, of a single leukocyte esterase dipstick compared to urine culture. [file 1471-2318-9-32-S1.doc]

| Table 1 - Test characteristics of a single leukocyte esterase dipstick compared to urine culture | | | | | | | | | |
| --- | --- | --- | --- | --- | --- | --- | --- | --- | --- |
|  |  |  | |  | |  | |  | |
|  |  |  | |  | |  | |  | |
|  |  | *Escherichia coli^a^* | | *Enterococcus faecalis^b^* | | *Klebsiella* species*^c^* | | Any bacteria*^d^* | |
|  |  | Visual reading*^e^* | Analyzer reading*^f^* | Visual reading*^e^* | Analyzer reading*^f^* | Visual reading*^e^* | Analyzer reading*^f^* | Visual reading*^e^* | Analyzer reading*^f^* |
|  |  |  |  |  |  |  |  |  |  |
|  |  |  |  |  |  |  |  |  |  |
| Sensitivity | >0 | 72% (64-79) | 81% (75-88) | 71% (49-92) | 82% (64-100) | 68% (50-86) | 71% (53-89) | 69% (62-75) | 78% (73-84) |
|  | >1 | 59% (51-67) | 62% (54-70) | 59% (35-82) | 65% (42-87) | 60% (41-79) | 58% (39-78) | 57% (50-64) | 61% (54-67) |
|  | >2 | 37% (29-45) | 38% (30-46) | 47% (23-71) | 41% (18-65) | 52% (32-72) | 46% (26-66) | 37% (30-44) | 38% (31-44) |
|  | >3 | 11% (6.1-17) | 17% (11-24) | 18% (0.0-36) | 29% (7.8-51) | 12% (0.0-25) | 25% (7.7-42) | 11% (6.5-15) | 18% (13-23) |
|  |  |  |  |  |  |  |  |  |  |
| Specificity | >0 | 61% (56-65) | 52% (48-57) | 54% (50-58) | 46% (42-50) | 54% (50-58) | 45% (42-49) | 64% (59-69) | 56% (51-60) |
|  | >1 | 74% (70-78) | 73% (69-76) | 67% (64-71) | 66% (62-69) | 68% (64-71) | 66% (62-69) | 78% (74-82) | 77% (73-81) |
|  | >2 | 84% (80-87) | 83% (80-86) | 80% (77-83) | 79% (76-82) | 80% (77-84) | 79% (76-82) | 87% (84-90) | 86% (83-89) |
|  | >3 | 96% (94-98) | 92% (89-94) | 94% (93-96) | 90% (88-93) | 94% (93-96) | 90% (88-93) | 96% (95-98) | 93% (91-96) |
|  |  |  |  |  |  |  |  |  |  |
| PPV | >0 | 34% (29-40) | 33% (28-38) | 4.1% (1.8-6.3) | 4.0% (1.9-6.0) | 5.8% (3.1-8.5) | 4.8% (2.6-7.0) | 48% (42-54) | 45% (40-51) |
|  | >1 | 40% (33-46) | 39% (33-46) | 4.8% (1.9-7.6) | 4.9% (2.1-7.7) | 7.1% (3.7-11) | 6.2% (3.1-9.3) | 56% (49-62) | 55% (49-62) |
|  | >2 | 39% (31-48) | 40% (31-48) | 6.1% (2.0-10) | 5.0% (1.4-8.7) | 9.9% (4.8-15) | 7.9% (3.4-12) | 58% (49-66) | 56% (48-64) |
|  | >3 | 43% (27-59) | 38% (26-50) | 8.1% (0.0-17) | 7.6% (1.2-14) | 8.1% (0.0-17) | 9.1% (2.2-16) | 59% (44-75) | 56% (44-68) |
|  |  |  |  |  |  |  |  |  |  |
| NPV | >0 | 88% (85-92) | 91% (87-94) | 99% (97-100) | 99% (98-100) | 98% (96-99) | 98% (96-99) | 81% (77-85) | 84% (80-89) |
|  | >1 | 86% (83-89) | 87% (84-90) | 98% (97-100) | 99% (97-100) | 98% (96-99) | 98% (96-99) | 79% (75-83) | 81% (77-84) |
|  | >2 | 82% (79-85) | 83% (79-86) | 98% (97-99) | 98% (97-99) | 98% (96-99) | 97% (96-99) | 74% (70-78) | 75% (71-78) |
|  | >3 | 79% (76-82) | 80% (76-83) | 98% (96-99) | 98% (97-99) | 96% (95-98) | 97% (95-98) | 69% (65-73) | 71% (67-74) |
|  |  |  |  |  |  |  |  |  |  |
|  |  |  |  |  |  |  |  |  |  |
| *^a^* 143 of 651 urine cultures showed growth of *Escherichia coli* | | | | | | | | | |
| *^b^* 17 of 651 urine cultures showed growth of *Enterococcus faecalis* | | | | | | | | | |
| *^c^* 25 of 651 urine cultures showed growth of *Klebsiella* spp*.* | | | | | | | | | |
| *^d^* 207 of 651 urine cultures showed growth of any bacteria. Any bacteria may be *E. coli, E. faecalis, Klebsiella* spp*., E. faecium, Enterobacter* spp*, coagulase-negative staphylococci, alfa-hemolytic streptococci, beta-hemolytic streptococci, Proteus mirabilis, P. vulgaris, Group B Streptococci* and *Pseudomonas aeruginosa.* | | | | | | | | | |
| *^e^* Number of visual readings: 630 | | | | | | | | | |
| *^f^* Number of analyzer readings: 642 | | | | | | | | | |
