# Supplementary material for: Evaluation of dipstick analysis among elderly residents to detect bacteriuria: a cross-sectional study in 32 nursing homes
Source: BMC Geriatr. 2009 Jul 27;9:32. doi: 10.1186/1471-2318-9-32 (PMC2724370; doi:10.1186/1471-2318-9-32)
Supplement: Additional file 2 — Table 2 – Test characteristics of a single nitrite dipstick compared to urine culture. Test characteristics, such as sensitivity, specificity, positive and negative predictive value, of a single nitrite dipstick compared to urine culture. [file 1471-2318-9-32-S2.doc]

| Table 2 - Test characteristics of a single nitrite dipstick compared to urine culture | | | | | | | | |
| --- | --- | --- | --- | --- | --- | --- | --- | --- |
|  |  | |  | |  | |  | |
|  |  | |  | |  | |  | |
|  | *Escherichia coli^a^* | | *Enterococcus faecalis^b^* | | *Klebsiella* species*^c^* | | Any bacteria*^d^* | |
|  | Visual reading*^e^* | Analyzer reading*^f^* | Visual reading*^e^* | Analyzer reading*^f^* | Visual reading*^e^* | Analyzer reading*^f^* | Visual reading*^e^* | Analyzer reading*^f^* |
|  |  |  |  |  |  |  |  |  |
|  |  |  |  |  |  |  |  |  |
|  |  |  |  |  |  |  |  |  |
| Sensitivity | 64% (56-72) | 62% (54-70) | 29% (7.8-51) | 29% (7.8-51) | 48% (28-68) | 50% (30-70) | 57% (50-63) | 56% (49-62) |
|  |  |  |  |  |  |  |  |  |
| Specificity | 88% (85-90) | 86% (83-89) | 76% (73-80) | 76% (73-79) | 77% (74-81) | 77% (73-80) | 92% (89-94) | 90% (88-93) |
|  |  |  |  |  |  |  |  |  |
| PPV | 59% (51-67) | 56% (49-64) | 3.3% (0.45-6.1) | 3.2% (0.44-6.0) | 7.8% (3.6-12) | 7.7% (3.5-12) | 76% (69-83) | 73% (66-80) |
|  |  |  |  |  |  |  |  |  |
| NPV | 90% (87-92) | 89% (86-92) | 98% (96-99) | 98% (96-99) | 97% (96-99) | 98% (96-99) | 82% (78-85) | 81% (78-85) |
|  |  |  |  |  |  |  |  |  |
|  |  |  |  |  |  |  |  |  |
| *^a^* 143 of 651 urine cultures showed growth of *Escherichia coli* | | | | | | | | |
| *^b^* 17 of 651 urine cultures showed growth of *Enterococcus faecalis* | | | | | | | | |
| *^c^* 25 of 651 urine cultures showed growth of *Klebsiella* spp*.* | | | | | | | | |
| *^d^* 207 of 651 urine cultures showed growth of any bacteria. Any bacteria may be *E. coli, E. faecalis, Klebsiella* spp*., E. faecium, Enterobacter* spp.*, coagulase-negative staphylococci, alfa-hemolytic streptococci, beta-hemolytic streptococci, Proteus mirabilis, P. vulgaris, Group B Streptococci* and *Pseudomonas aeruginosa.* | | | | | | | | |
| *^e^* Number of visual readings: 650 | | | | | | | | |
| *^f^* Number of analyzer readings: 643 | | | | | | | | |
