# Supplementary material for: Evaluation of dipstick analysis among elderly residents to detect bacteriuria: a cross-sectional study in 32 nursing homes
Source: BMC Geriatr. 2009 Jul 27;9:32. doi: 10.1186/1471-2318-9-32 (PMC2724370; doi:10.1186/1471-2318-9-32)
Supplement: Additional file 3 — Table 3 – Test characteristics of a positive leukocyte esterase and a positive nitrite dipstick compared to urine culture. Test characteristics, such as sensitivity, specificity, positive and negative predictive value, of a positive leukocyte esterase and a positive nitrite dipstick compared to urine culture. [file 1471-2318-9-32-S3.doc]

| Table 3 - Test characteristics of a positive leukocyte esterase and a positive nitrite dipstick compared to urine culture | | | | | | | | | |
| --- | --- | --- | --- | --- | --- | --- | --- | --- | --- |
|  |  |  | |  | |  | |  | |
|  |  |  | |  | |  | |  | |
|  |  | *Escherichia coli^a^* | | *Enterococcus faecalis^b^* | | *Klebsiella* species*^c^* | | Any bacteria*^d^* | |
|  |  | Visual reading*^e^* | Analyzer reading*^f^* | Visual reading*^e^* | Analyzer reading*^f^* | Visual reading*^e^* | Analyzer reading*^f^* | Visual reading*^e^* | Analyzer reading*^f^* |
|  |  |  |  |  |  |  |  |  |  |
|  |  |  |  |  |  |  |  |  |  |
| Sensitivity | >0 | 50% (42-59) | 55% (47-63) | 18% (0.0-36) | 24% (3.4-44) | 36% (17-55) | 42% (22-61) | 44% (37-51) | 48% (41-55) |
|  | >1 | 42% (34-50) | 45% (37-53) | 18% (0.0-36) | 18% (0.0-36) | 32% (14-50) | 33% (14-52) | 37% (30-44) | 40% (33-46) |
|  | >2 | 25% (18-32) | 27% (19-34) | 12% (0.0-27) | 12% (0.0-27) | 28% (10-46) | 29% (11-47) | 23% (18-29) | 25% (19-31) |
|  | >3 | 8.5% (3.9-13) | 12% (6.6-17) | 0.0% (0.0-0.0) | 5.9% (0.0-17) | 8.0% (0.0-19) | 13% (0.0-26) | 7.3% (3.8-11) | 11% (6.9-16) |
|  |  |  |  |  |  |  |  |  |  |
| Specificity | >0 | 91% (88-94) | 89% (87-92) | 82% (79-85) | 80% (77-83) | 82% (79-86) | 80% (77-84) | 94% (92-96) | 93% (90-95) |
|  | >1 | 94% (92-96) | 93% (91-95) | 86% (83-89) | 85% (82-87) | 86% (84-89) | 85% (82-88) | 97% (95-98) | 96% (94-98) |
|  | >2 | 95% (93-97) | 95% (94-97) | 91% (88-93) | 90% (88-93) | 91% (89-93) | 91% (89-93) | 97% (96-99) | 98% (96-99) |
|  | >3 | 99% (98-100) | 98% (96-99) | 97% (96-98) | 96% (94-97) | 97% (96-99) | 96% (94-97) | 99% (98-100) | 99% (98-100) |
|  |  |  |  |  |  |  |  |  |  |
| PPV | >0 | 62% (53-71) | 60% (52-68) | 2.6% (0.0-5.5) | 3.1% (0.1-6.1) | 7.8% (2.9-13) | 7.7% (3.1-12) | 78% (71-86) | 76% (69-83) |
|  | >1 | 66% (56-75) | 65% (55-74) | 3.3% (0.0-7.0) | 3.0% (0.0-6.4) | 8.9% (3.0-15) | 8.1% (2.7-13) | 84% (77-92) | 82% (74-89) |
|  | >2 | 58% (46-71) | 62% (50-74) | 3.3% (0.0-7.9) | 3.3% (0.0-7.8) | 12% (3.5-20) | 11% (3.5-19) | 80% (70-90) | 84% (74-93) |
|  | >3 | 63% (41-85) | 61% (43-79) | 0.0% (0.0-0.0) | 3.6% (0.0-10) | 11% (0.0-24) | 11% (0.0-22) | 79% (61-97) | 82% (68-96) |
|  |  |  |  |  |  |  |  |  |  |
| NPV | >0 | 86% (83-89) | 87% (84-90) | 97% (96-99) | 97% (96-99) | 97% (95-98) | 97% (96-99) | 78% (74-81) | 79% (76-83) |
|  | >1 | 85% (82-88) | 86% (83-88) | 97% (96-99) | 97% (96-99) | 97% (95-98) | 97% (96-98) | 76% (73-80) | 77% (73-81) |
|  | >2 | 81% (78-85) | 82% (79-85) | 97% (96-99) | 97% (96-99) | 97% (95-98) | 97% (96-98) | 72% (69-76) | 73% (70-77) |
|  | >3 | 79% (76-82) | 79% (76-83) | 97% (96-99) | 97% (96-99) | 96% (95-98) | 97% (95-98) | 69% (65-73) | 70% (66-74) |
|  |  |  |  |  |  |  |  |  |  |
|  |  |  |  |  |  |  |  |  |  |
| *^a^* 143 of 651 urine cultures showed growth of *Escherichia coli* | | | | | | | | | |
| *^b^* 17 of 651 urine cultures showed growth of *Enterococcus faecalis* | | | | | | | | | |
| *^c^* 25 of 651 urine cultures showed growth of *Klebsiella* spp*.* | | | | | | | | | |
| *^d^* 207 of 651 urine cultures showed growth of any bacteria. Any bacteria may be *E. coli, E. faecalis, Klebsiella* spp*., E. faecium, Enterobacter* spp.*, coagulase-negative staphylococci, alfa-hemolytic streptococci, beta-hemolytic streptococci, Proteus mirabilis, P. vulgaris, Group B Streptococci* and *Pseudomonas aeruginosa.* | | | | | | | | | |
| *^e^* Number of visual readings: 630 | | | | | | | | | |
| *^f^* Number of analyzer readings: 637 | | | | | | | | | |
