# Supplementary material for: Evaluation of dipstick analysis among elderly residents to detect bacteriuria: a cross-sectional study in 32 nursing homes
Source: BMC Geriatr. 2009 Jul 27;9:32. doi: 10.1186/1471-2318-9-32 (PMC2724370; doi:10.1186/1471-2318-9-32)
Supplement: Additional file 4 — Table 4 – Test characteristics of a positive leukocyte esterase and/or a positive nitrite dipstick compared to urine culture. Test characteristics, such as sensitivity, specificity, positive and negative predictive value, of a positive leukocyte esterase and/or a positive nitrite dipstick compared to urine culture. [file 1471-2318-9-32-S4.doc]

| Table 4 - Test characteristics of a positive leukocyte esterase and/or a positive nitrite dipstick compared to urine culture | | | | | | | | | |
| --- | --- | --- | --- | --- | --- | --- | --- | --- | --- |
|  |  |  | |  | |  | |  | |
|  |  |  | |  | |  | |  | |
|  |  | *Escherichia coli^a^* | | *Enterococcus faecalis^b^* | | *Klebsiella* species*^c^* | | Any bacteria*^d^* | |
|  |  | Visual reading*^e^* | Analyzer reading*^f^* | Visual reading*^e^* | Analyzer reading*^f^* | Visual reading*^e^* | Analyzer reading*^f^* | Visual reading*^e^* | Analyzer reading*^f^* |
|  |  |  |  |  |  |  |  |  |  |
|  |  |  |  |  |  |  |  |  |  |
| Sensitivity | >0 | 86% (80-92) | 88% (83-93) | 82% (64-100) | 88% (73-100) | 80% (64-96) | 79% (63-95) | 82% (77-87) | 85% (81-90) |
|  | >1 | 82% (75-88) | 80% (73-86) | 71% (49-92) | 76% (56-97) | 76% (59-93) | 75% (58-92) | 77% (71-83) | 77% (71-83) |
|  | >2 | 77% (70-84) | 74% (67-81) | 65% (42-87) | 59% (35-82) | 72% (54-90) | 67% (48-86) | 71% (65-77) | 69% (62-75) |
|  | >3 | 67% (60-75) | 68% (60-75) | 47% (23-71) | 53% (29-77) | 52% (32-72) | 63% (43-82) | 60% (54-67) | 62% (56-69) |
|  |  |  |  |  |  |  |  |  |  |
| Specificity | >0 | 57% (52-61) | 49% (44-53) | 48% (44-52) | 41% (38-45) | 48% (44-52) | 41% (38-45) | 61% (57-66) | 53% (48-58) |
|  | >1 | 68% (64-72) | 66% (62-70) | 57% (54-61) | 57% (53-61) | 58% (54-62) | 57% (53-61) | 73% (69-77) | 71% (67-76) |
|  | >2 | 76% (72-80) | 74% (70-78) | 65% (61-69) | 64% (60-68) | 66% (62-70) | 65% (61-68) | 81% (77-85) | 79% (75-83) |
|  | >3 | 84% (81-88) | 80% (77-84) | 73% (70-77) | 70% (67-74) | 74% (70-77) | 71% (67-74) | 89% (86-92) | 85% (81-88) |
|  |  |  |  |  |  |  |  |  |  |
| PPV | >0 | 36% (31-42) | 33% (28-38) | 4.2% (2.1-6.4) | 4.0% (2.0-5.9) | 6.0% (3.5-8.6) | 5.0% (2.8-7.2) | 51% (45-56) | 46% (41-51) |
|  | >1 | 42% (36-48) | 40% (34-46) | 4.4% (2.0-6.8) | 4.6% (2.2-7.1) | 7.0% (3.9-10) | 6.4% (3.5-9.2) | 58% (52-64) | 56% (50-62) |
|  | >2 | 48% (41-55) | 45% (39-51) | 4.9% (2.1-7.7) | 4.3% (1.7-6.9) | 8.0% (4.5-12) | 6.9% (3.6-10) | 64% (58-71) | 61% (54-67) |
|  | >3 | 56% (48-63) | 49% (42-57) | 4.7% (1.5-7.8) | 4.6% (1.7-7.6) | 7.6% (3.6-12) | 7.7% (4.0-11) | 73% (66-79) | 66% (59-73) |
|  |  |  |  |  |  |  |  |  |  |
| NPV | >0 | 93% (90-96) | 93% (90-96) | 99% (98-100) | 99% (98-100) | 98% (97-100) | 98% (96-100) | 88% (84-91) | 88% (85-92) |
|  | >1 | 93% (90-95) | 92% (89-95) | 99% (97-100) | 99% (98-100) | 98% (97-100) | 98% (97-100) | 87% (83-90) | 87% (83-90) |
|  | >2 | 92% (89-95) | 91% (88-94) | 99% (97-100) | 98% (97-100) | 98% (97-100) | 98% (97-99) | 85% (82-89) | 84% (81-88) |
|  | >3 | 90% (87-93) | 90% (87-92) | 98% (97-99) | 98% (97-99) | 97% (96-99) | 98% (97-99) | 82% (79-86) | 83% (79-86) |
|  |  |  |  |  |  |  |  |  |  |
|  |  |  |  |  |  |  |  |  |  |
| *^a^* 143 of 651 urine cultures showed growth of *Escherichia coli* | | | | | | | | | |
| *^b^* 17 of 651 urine cultures showed growth of *Enterococcus faecalis* | | | | | | | | | |
| *^c^* 25 of 651 urine cultures showed growth of *Klebsiella* spp*.* | | | | | | | | | |
| *^d^* 207 of 651 urine cultures showed growth of any bacteria. Any bacteria may be *E. coli, E. faecalis, Klebsiella* spp*., E. faecium, Enterobacter* spp.*, coagulase-negative staphylococci, alfa-hemolytic streptococci, beta-hemolytic streptococci, Proteus mirabilis, P. vulgaris, Group B Streptococci* and *Pseudomonas aeruginosa.* | | | | | | | | | |
| *^e^* Number of visual readings: 630 | | | | | | | | | |
| *^f^* Number of analyzer readings: 637 | | | | | | | | | |
