# Supplementary material for: Evaluation of dipstick analysis among elderly residents to detect bacteriuria: a cross-sectional study in 32 nursing homes
Source: BMC Geriatr. 2009 Jul 27;9:32. doi: 10.1186/1471-2318-9-32 (PMC2724370; doi:10.1186/1471-2318-9-32)
Supplement: Additional file 5 — Table 5 – Odds ratio for a positive dipstick to predict presence of bacteriuria when considering age and sex. Odds ratio for a positive dipstick to predict presence of bacteriuria when considering age and sex. [file 1471-2318-9-32-S5.doc]

| Table 5 - Odds ratio for a positive dipstick to predict presence of bacteriuria when considering age and sex | | | | | | | | | |
| --- | --- | --- | --- | --- | --- | --- | --- | --- | --- |
|  |  |  | |  | |  | |  | |
|  |  |  | |  | |  | |  | |
|  |  | *Escherichia coli^a^* | | *Enterococcus faecalis^b^* | | *Klebsiella* species*^c^* | | Any bacteria*^d^* | |
|  |  | Visual reading*^e^* | Analyzer reading*^f^* | Visual reading*^e^* | Analyzer reading*^f^* | Visual reading*^e^* | Analyzer reading*^f^* | Visual reading*^e^* | Analyzer reading*^f^* |
|  |  |  |  |  |  |  |  |  |  |
|  |  |  |  |  |  |  |  |  |  |
| Leukocyte | >0 | 3.3 (2.1-4.9) --- <0.001 | 3.7 (2.3-5.9) --- <0.001 | 3.3 (1.1-9.8) --- 0.033 | 4.7 (1.3-17) --- 0.020 | 2.1 (0.90-5.1) --- 0.087 | 1.6 (0.65-4.1) --- 0.30 | 3.4 (2.4-4.9) --- <0.001 | 3.8 (2.5-5.6) --- <0.001 |
| esterase | >1 | 3.6 (2.4-5.4) --- <0.001 | 3.9 (2.6-5.9) --- <0.001 | 3.3 (1.2-9.2) --- 0.020 | 3.7 (1.3-10) --- 0.012 | 2.8 (1.2-6.4) --- 0.014 | 2.4 (1.0-5.6) --- 0.038 | 4.3 (3.0-6.2) --- <0.001 | 4.7 (3.3-6.8) --- <0.001 |
|  | >2 | 2.7 (1.8-4.2) --- <0.001 | 2.7 (1.8-4.2) --- <0.001 | 3.7 (1.4-10) --- 0.0094 | 2.8 (1.0-7.6) --- 0.048 | 4.1 (1.8-9.3) --- <0.001 | 2.9 (1.3-6.8) --- 0.011 | 3.7 (2.4-5.5) --- <0.001 | 3.4 (2.3-5.1) --- <0.001 |
|  | >3 | 2.8 (1.4-5.7) --- 0.0043 | 2.0 (1.2-3.5) --- 0.014 | 3.7 (1.0-14) --- 0.050 | 3.9 (1.3-12) --- 0.016 | 2.2 (0.63-7.8) --- 0.22 | 2.9 (1.1-7.7) --- 0.034 | 3.3 (1.6-6.6) --- <0.001 | 2.7 (1.6-4.6) --- <0.001 |
|  |  |  |  |  |  |  |  |  |  |
| Nitrite | Pos | 12 (7.5-18) --- <0.001 | 9.8 (6.3-15) --- <0.001 | 1.4 (0.49-4.2) --- 0.52 | 1.4 (0.48-4.1) --- 0.54 | 2.8 (1.2-6.2) --- 0.015 | 2.9 (1.3-6.7) --- 0.012 | 14 (8.7-21) --- <0.001 | 11 (7.3-17) --- <0.001 |
|  |  |  |  |  |  |  |  |  |  |
|  |  |  |  |  |  |  |  |  |  |
| *^a^* 143 of 651 urine cultures showed growth of *Escherichia coli* | | | | | | | | | |
| *^b^* 17 of 651 urine cultures showed growth of *Enterococcus faecalis* | | | | | | | | | |
| *^c^* 25 of 651 urine cultures showed growth of *Klebsiella* spp*.* | | | | | | | | | |
| *^d^* 207 of 651 urine cultures showed growth of any bacteria. Any bacteria may be *E. coli, E. faecalis, Klebsiella* spp*., E. faecium, Enterobacter* spp.*, coagulase-negative staphylococci, alfa-hemolytic streptococci, beta-hemolytic streptococci, Proteus mirabilis, P. vulgaris, Group B Streptococci* and *Pseudomonas aeruginosa.* | | | | | | | | | |
| *^e^* Number of visual readings for leukocyte esterase were 630 and 650 for nitrite | | | | | | | | | |
| *^f^* Number of analyzer readings for leukocyte esterase were 642 and 643 for nitrite | | | | | | | | | |
